# Supplementary material for: Rapalink-1 reveals TOR-dependent genes and an agmatinergic axis-based metabolic feedback regulating TOR activity and lifespan in fission yeast
Source: Commun Biol. 2025 Sep 29;8:1364. doi: 10.1038/s42003-025-08731-3 (PMC12479844; doi:10.1038/s42003-025-08731-3)
Supplement: Supplementary file 2 — Supplementary Information [file 42003_2025_8731_MOESM2_ESM.pdf]

Supplemental Figure 1

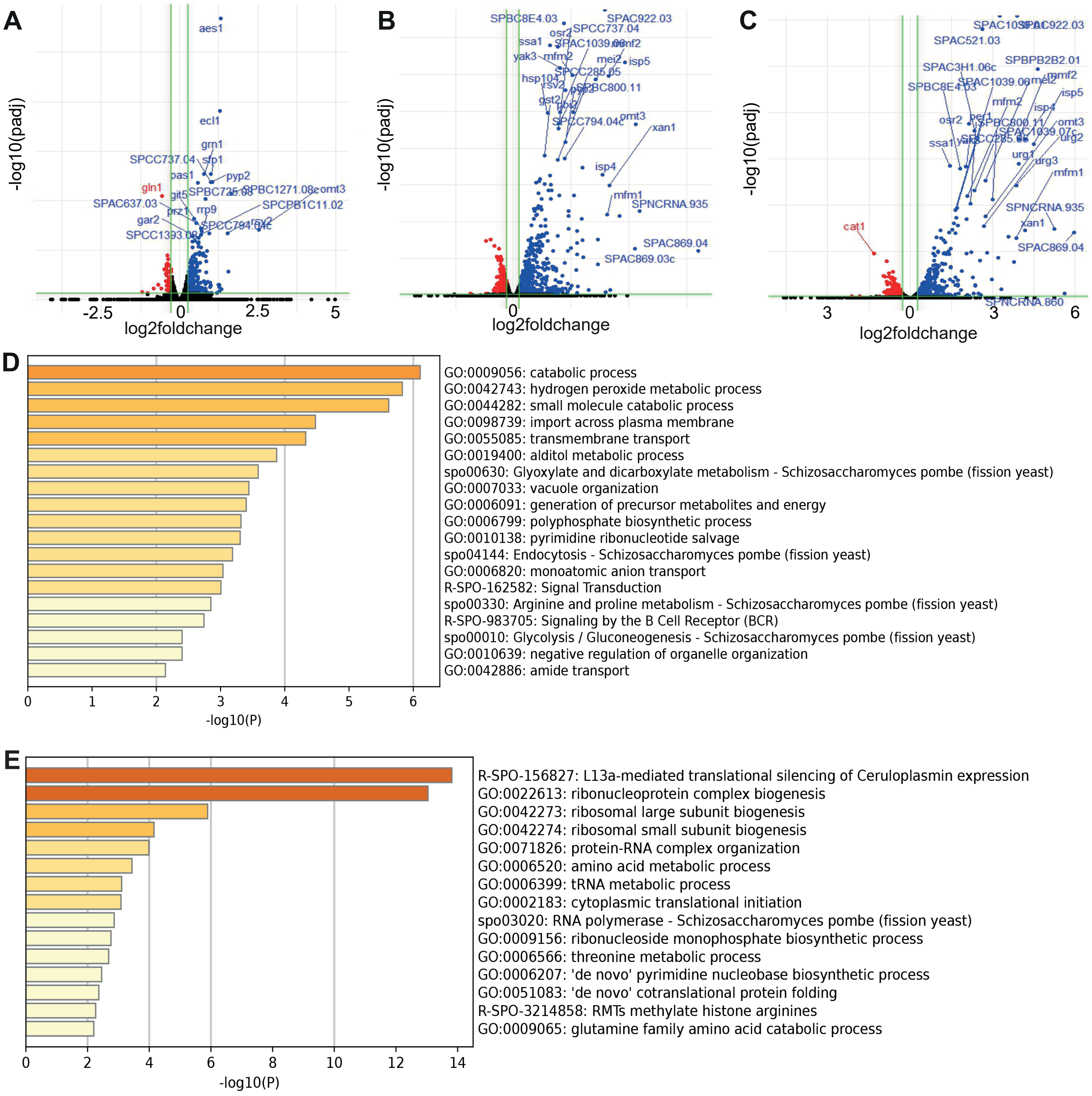

Supplemental Figure 1. RNAseq data analyses of the paper related to Figure 3.
(A-C) Volcano plots with upregulated (red), downregulated (blue) and unchanged (black) genes.
(A) Untreated versus rapamycin-treated cells.
(B) Untreated versus rapalink-1-treated cells.
(C) rapamycin versus rapalink-1-treated cells.
(D) Bar graph representation of representative GO ontology enrichments for genes upregulated in rapalink-1 treatment only (related to Figure 3E)
(E) Bar graph representation of representative GO ontology enrichments for genes downregulated in rapalink-1 treatment only (related to Figure 3F).

Supplemental Figure 2

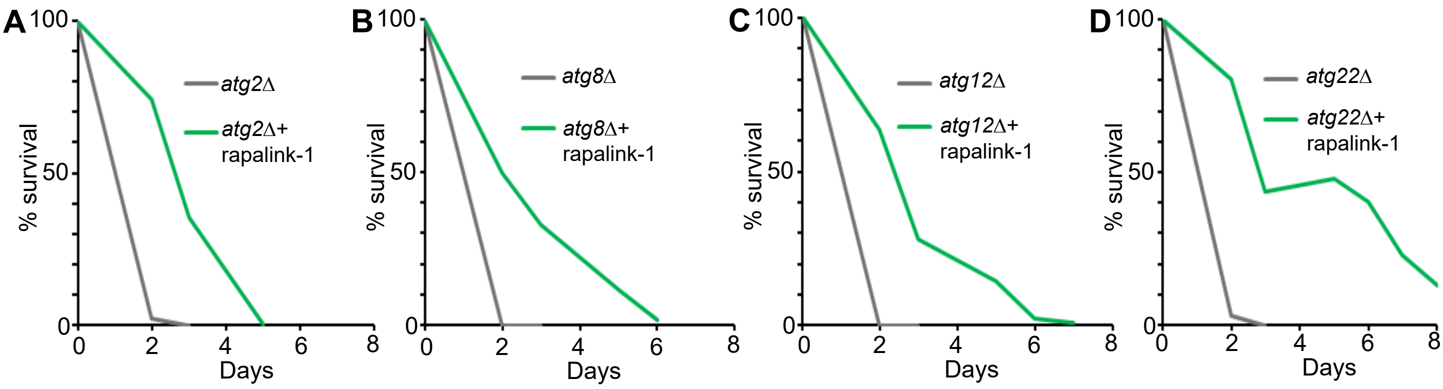

Supplemental Figure 2. Rapalink-1 supplementation extends lifespans of atg mutants.
(A) CLS assay for atg2Δ with and without rapalink-1 as indicated.
(B) CLS assay for atg8Δ with and without rapalink-1 as indicated.
(C) CLS assay for atg12Δ with and without rapalink-1 as indicated.
(D) CLS assay for atg22Δ with and without rapalink-1 as indicated.
In all cases the lifespan extension is statistically significant (log rank $p<0.01$).

Supplemental Figure 3

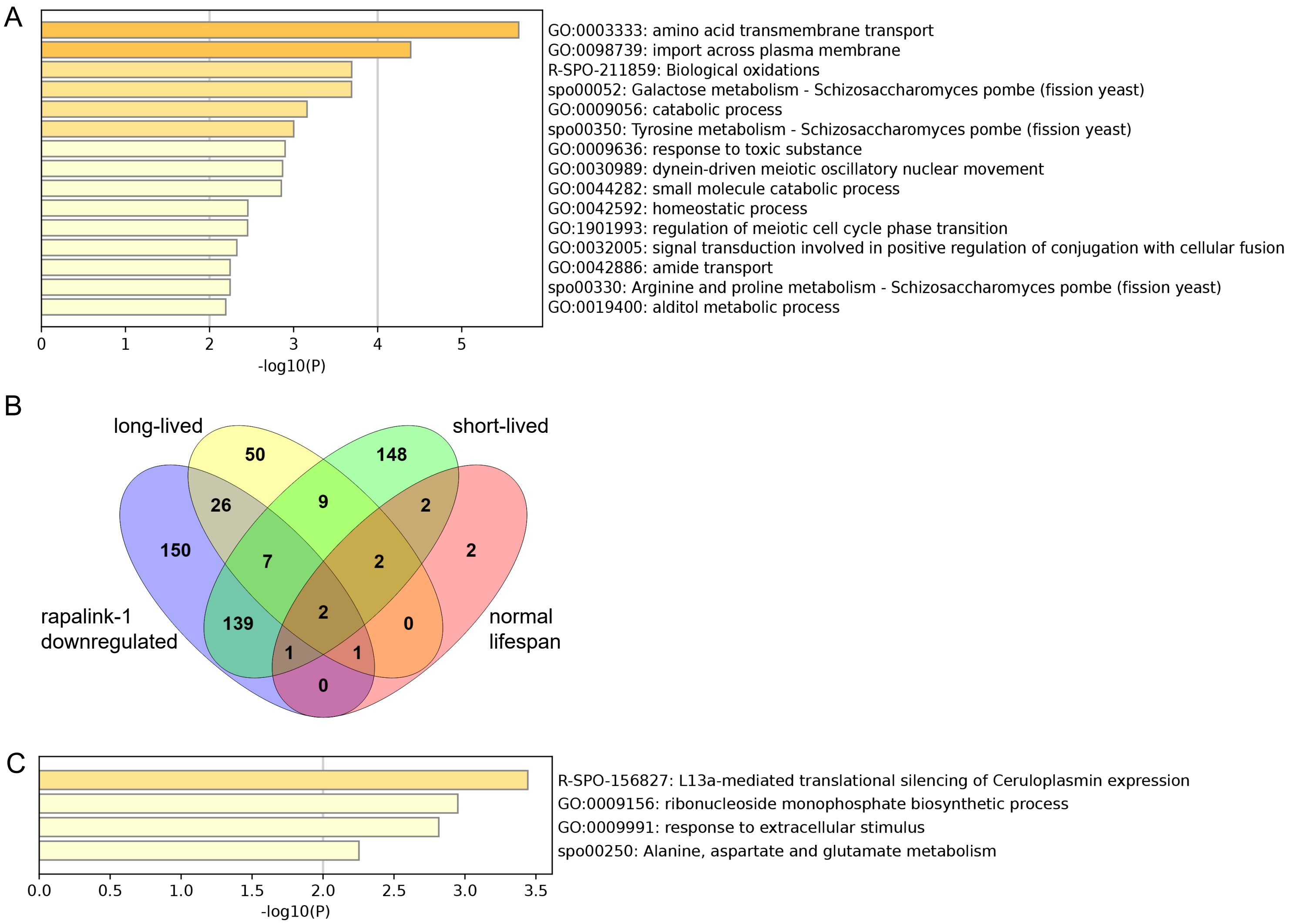

Supplemental Figure 3. Gene expression analyses and enrichments related to rapalink-1.
(A) Bar graph schematic for representative GO ontology enrichments of rapalink-1-upregulated genes not related to lifespan phenotype (related to Figure 2H, genes in red fonts).
(B) Venn diagram showing overlaps of rapalink-1 downregulated genes (the portion that corresponding mutants are viable) with genes that their deletions lead to long-lived, short-lived or normal-lived mutants.
(C) Bar graph schematic for representative GO ontology enrichments of the 150 rapalink-1-downregulated genes not related to lifespan phenotype shown in (B).

Supplemental Figure 4

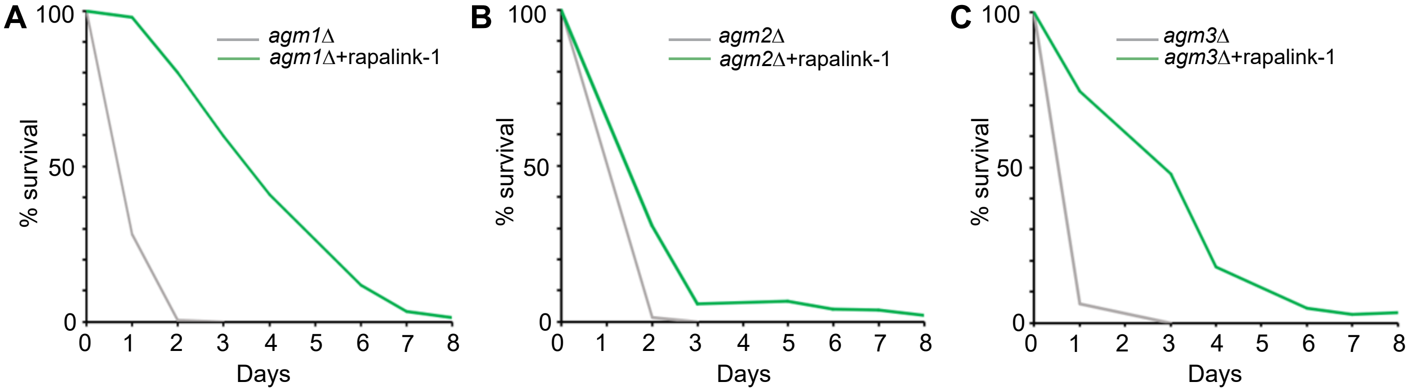

Supplemental Figure 4. Rapalink-1 supplementation extends lifespans of agmatinase mutants.
(A) CLS assay for agm1Δ with and without rapalink-1 as indicated.
(B) CLS assay for agm2Δ with and without rapalink-1 as indicated.
(C) CLS assay for agm3Δ with and without rapalink-1 as indicated.
In all cases the lifespan extension is statistically significant (log rank $p<0.01$).

Supplemental Figure 5

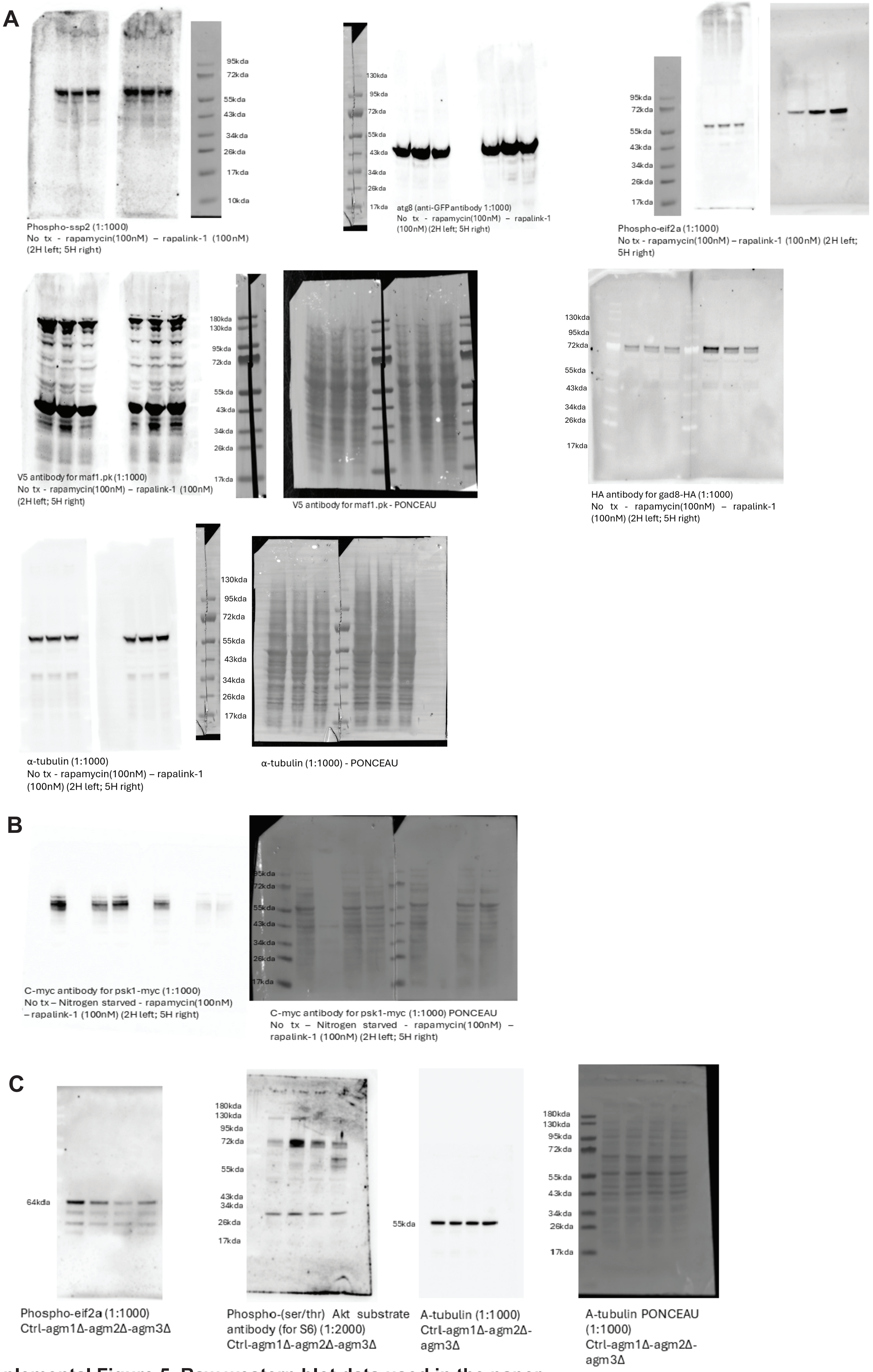

Supplemental Figure 5. Raw western blot data used in the paper.
(A) Western blots for main figure 1E.
(B) Western blots for main figure 1F.
(C) Western blots for main figure 5G.
