# Supplementary material for: Rapalink-1 reveals TOR-dependent genes and an agmatinergic axis-based metabolic feedback regulating TOR activity and lifespan in fission yeast
Source: Commun Biol. 2025 Sep 29;8:1364. doi: 10.1038/s42003-025-08731-3 (PMC12479844; doi:10.1038/s42003-025-08731-3)
Supplement: Supplementary file 3 — description of additional supplementary files [file 42003_2025_8731_MOESM3_ESM.pdf]

Description of Additional Supplementary Files

File name: Supplementary Data 1

Description: Numerical data used for the generation of lifespans and qPCR graphs in the manuscript.

File name: Supplementary Data 2

Description: List of rapalink-1 sensitive (<0.75, representing 25% smaller colony compared to untreated control) and resistant (>1.25, 25% larger colony compared to untreated control) mutant strains.rapalink-1 sensitive (<0.75) and resistant (>1.25) strains.

File name: Supplementary Data 3

Description: List of genes upregulated in fast growing fission yeast cells (0.5 OD600) following 5 hrs of 100nM rapamycin treatment in YES media.

File name: Supplementary Data 4

Description: List of genes downregulated in fast growing fission yeast cells (0.5 OD600) following 5 hrs of 100nM rapamycin treatment in YES media.

File name: Supplementary Data 5

Description: List of genes upregulated in fast growing fission yeast cells (0.5 OD600) following 5 hrs of 100nM rapalink-1 treatment in YES media.

File name: Supplementary Data 6

Description: List of genes downregulated in fast growing fission yeast cells (0.5 OD600) following 5 hrs of 100nM rapalink-1 treatment in YES media.

File name: Supplementary Data 7

Description: Fitness ratio values of mutant strains following genome-wide phenomics screen with Agmatine and Putrescine (see main text and materials and methods).

File name: Supplementary Data 8

Description: Interaction values for all mutant strains against agm1 following normalizations (see Materials and Methods) and filtering of absent colonies, control and experimental SGA linkage, high variability of colonies and low fitness strains.
