# Supplementary material for: Rapalink-1 reveals TOR-dependent genes and an agmatinergic axis-based metabolic feedback regulating TOR activity and lifespan in fission yeast
Source: Commun Biol. 2025 Sep 29;8:1364. doi: 10.1038/s42003-025-08731-3 (PMC12479844; doi:10.1038/s42003-025-08731-3)
Supplement: Supplementary file 12 — Reporting summary [file 42003_2025_8731_MOESM12_ESM.pdf]

nature portfolio

Corresponding author(s): Charalampos Rallis

Last updated by author(s): Aug 9, 2025

Reporting Summary

Nature Portfolio wishes to improve the reproducibility of the work that we publish. This form provides structure for consistency and transparency in reporting. For further information on Nature Portfolio policies, see our Editorial Policies and the Editorial Policy Checklist.

Statistics

For all statistical analyses, confirm that the following items are present in the figure legend, table legend, main text, or Methods section.

n/a	Confirmed	
☐	☒	The exact sample size (*n*) for each experimental group/condition, given as a discrete number and unit of measurement
☐	☒	A statement on whether measurements were taken from distinct samples or whether the same sample was measured repeatedly
☐	☒	The statistical test(s) used AND whether they are one- or two-sided<br>*Only common tests should be described solely by name; describe more complex techniques in the Methods section.*
☐	☒	A description of all covariates tested
☐	☒	A description of any assumptions or corrections, such as tests of normality and adjustment for multiple comparisons
☐	☒	A full description of the statistical parameters including central tendency (e.g. means) or other basic estimates (e.g. regression coefficient) AND variation (e.g. standard deviation) or associated estimates of uncertainty (e.g. confidence intervals)
☐	☒	For null hypothesis testing, the test statistic (e.g. *F*, *t*, *r*) with confidence intervals, effect sizes, degrees of freedom and *P* value noted<br>*Give P values as exact values whenever suitable.*
☒	☐	For Bayesian analysis, information on the choice of priors and Markov chain Monte Carlo settings
☒	☐	For hierarchical and complex designs, identification of the appropriate level for tests and full reporting of outcomes
☐	☒	Estimates of effect sizes (e.g. Cohen's *d*, Pearson's *r*), indicating how they were calculated

Our web collection on statistics for biologists contains articles on many of the points above.

Software and code

Policy information about availability of computer code

Data collection	Data are collected through experimental approaches and methods as described in the manuscript.
Data analysis	RStudio 2023.06.2+561; ImageJ 1.53t; Metascape (https://metascape.org/gp/index.html#/main/step1)

For manuscripts utilizing custom algorithms or software that are central to the research but not yet described in published literature, software must be made available to editors and reviewers. We strongly encourage code deposition in a community repository (e.g. GitHub). See the Nature Portfolio guidelines for submitting code & software for further information.

Data

Policy information about availability of data

All manuscripts must include a data availability statement. This statement should provide the following information, where applicable:

- Accession codes, unique identifiers, or web links for publicly available datasets
- A description of any restrictions on data availability
- For clinical datasets or third party data, please ensure that the statement adheres to our policy

RNA-seq data are deposited and are available from GEO (accession number: GSE272269). The Numerical data used for the generation of lifespans and qPCR graphs in the manuscript provided in Supplementary Data 1. Processed omics data (RNA-seq, genome-wide drug screens and Synthetic Genetic Array screens are all provided in Supplementary Data 2-8. All yeast strains are available from the corresponding author (or other sources/laboratories that have been generated in) on reasonable request. Uncropped western blot images are provided into the Supplementary Information (Supplemental Figure 5).

April 2023

Research involving human participants, their data, or biological material

Policy information about studies with human participants or human data. See also policy information about sex, gender (identity/presentation), and sexual orientation and race, ethnicity and racism.

Reporting on sex and gender	n/a
Reporting on race, ethnicity, or other socially relevant groupings	n/a
Population characteristics	n/a
Recruitment	n/a
Ethics oversight	n/a

Note that full information on the approval of the study protocol must also be provided in the manuscript.

Field-specific reporting

Please select the one below that is the best fit for your research. If you are not sure, read the appropriate sections before making your selection.

☒ Life sciences ☐ Behavioural & social sciences ☐ Ecological, evolutionary & environmental sciences

For a reference copy of the document with all sections, see nature.com/documents/nr-reporting-summary-flat.pdf

Life sciences study design

All studies must disclose on these points even when the disclosure is negative.

Sample size	Sample sizes are determined according to established practices in the field e.g. measurement of 200 septated cells for septation index; ; 50-100 cells for determination of cell size upon divisionat least 2 biological repeats with 2 technical repeats for each for lifespan data points.
Data exclusions	Small colony measurements signifying absent strains from our screens have been filtered out to avoid reporting false hits in compound screening and genetic interactions. This is a usual practice in the field.
Replication	Compound screening has been performed in densities to allow measuremtnts of quadruplicates for each strain. Lifespan assays have been performed in different weeks. Western blot repeats are performed in different weeks with material independently collected. Microscopy measurements involve adequate number of indivisual cells accordign to published practices in the field.
Randomization	Sample allocation is not random as it involves defined fission yeast mutant strains with specific and controlled compound/drug treatments.
Blinding	See randomisation. We analyse specific fission yeast strains and mutants with defined drug/compound treatments and in appropriate biological and technical replicates.

Reporting for specific materials, systems and methods

We require information from authors about some types of materials, experimental systems and methods used in many studies. Here, indicate whether each material, system or method listed is relevant to your study. If you are not sure if a list item applies to your research, read the appropriate section before selecting a response.

Materials & experimental systems

n/a	Involved in the study	
☐	☒	Antibodies
☒	☐	Eukaryotic cell lines
☒	☐	Palaeontology and archaeology
☒	☐	Animals and other organisms
☒	☐	Clinical data
☒	☐	Dual use research of concern
☒	☐	Plants

Methods

n/a	Involved in the study	
☒	☐	ChIP-seq
☒	☐	Flow cytometry
☒	☐	MRI-based neuroimaging

Antibodies

Antibodies used

Antibodies directed against phospho-eIF2a (#9721, 1:1,000), eIF2a (#9722, 1:1,000), phospho-Ssp2 (#50081, 1:1,000), Phospho-(Ser/Thr) Akt Substrate Antibody (#9611, 1:2,000) were purchased from Cell Signaling Technologies. The antibody directed against myc (ab32, 1:1,000) was purchased from Abcam while antibodies directed against V5 (sc-81594, 1:1,000) and HA (sc-7392, 1:1,000) were purchased from Santa Cruz Biotechnology. Secondary antibodies Goat Anti-Mouse IgG H&L (HRP) (ab205719, 1:5,000 in all cases except when V5 and GFP was the primary antibody where it was used at 1:10,000) and Goat Anti-Rabbit IgG H&L (HRP) (ab205718, 1:5,000) were purchased from Abcam while the ECL Western Blotting Detection system was from Pierce™. α-tubulin (T5168, 1:1,000) and anti-GFP (11814460001, 1:1,000) were purchased from Sigma-Aldrich. α-tubulin and Ponceau S staining have been used as loading controls for western blots as and where indicated. Western blotting quantifications have been performed using ImageJ/Fiji

Validation

WB validations:
#9721: validated in PC12 and C2C12 cells;
#9722: validated in PC12 cells;
#50081: validated in several cell lines including K-562, 293T, C2C12, C6, NCI-H2228
#9611: validated in Jurkat, A431, C2C12 and widely used used in the fission yeast field
#sc-81594: validated in 293T, against V5 tag widely used
#sc-7392: validated in COS cells, against HA-tag widely used
#T5168: validated in several systems including human fibroblasts and C. elegans, widely used, ascites fluid, clone B-5-1-2
#11814460001: against GFP, widely used antibody, from mouse IgG1κ (clones 7.1 and 13.1)
#ab32: validated in several systems includingJurkat and SH-SY5Y

Plants

Seed stocks

n/a

Novel plant genotypes

n/a

Authentication

n/a
